# MGM as a Large‐Scale Pretrained Foundation Model for Microbiome Analyses in Diverse Contexts

**DOI:** 10.1002/advs.202513333

**Published:** 2026-01-25

**Authors:** Haohong Zhang, Yuli Zhang, Zixin Kang, Jiayun Xiong, Ronghua Yang, Kang Ning

**Affiliations:** ^1^ Key Laboratory of Molecular Biophysics of the Ministry of Education, Hubei Key Laboratory of Bioinformatics and Molecular‐imaging, Center of AI Biology, Department of Bioinformatics and Systems Biology, College of Life Science and Technology Huazhong University of Science and Technology Wuhan Hubei China; ^2^ Dovetree Synbio Ltd. Shenyang China

**Keywords:** deep learning, foundation model, microbiome

## Abstract

Microbial communities are integral to human health, biotechnology, and environmental systems, yet their analysis is hindered by data heterogeneity and batch effects across studies. Traditional supervised methods often fail to capture universal patterns, limiting their utility in diverse contexts. Here, we present the Microbial General Model (MGM), the first large‐scale foundation model for microbiome analysis, pretrained on 260,000 samples using transformer‐based language modeling. MGM employs self‐attention mechanisms and autoregressive pre‐training to learn contextualized representations of microbial compositions, enabling robust transfer learning for downstream tasks. Benchmark evaluations demonstrate MGM's superior performance over conventional methods (average ROC‐AUC = 0.99 vs. 0.68–0.97) in microbial community classification, with enhanced generalization across geographic regions. MGM also captures spatial and temporal microbial dynamics, as evidenced by its application to a longitudinal infant cohort, where it delineated delivery mode‐specific microbiome trajectories and identified keystone genera such as *Bacteroides* and *Bifidobacterium* in vaginal deliveries and *Haemophilus* in cesarean deliveries. Furthermore, through prompt‐guided generation, MGM produced realistic microbial profiles conditioned on disease labels. By integrating self‐supervised learning with domain‐specific fine‐tuning, MGM advances the scalability and precision of microbiome analyses, offering a unified framework for diagnostics, ecological studies, and therapeutic discovery.

## Graphical abstract

We present the Microbial General Model (MGM), a transformer‐based foundation model pretrained on over 260,000 microbiome samples. MGM learns contextualized microbial representations via self‐supervised language modeling, enabling robust transfer learning, cross‐regional generalization, keystone taxa discovery, and prompt‐guided generation of realistic, disease‐specific microbiome profiles across diverse biological and clinical contexts.

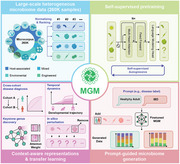

## 1 Introduction

Microbial communities, ubiquitous across diverse environments, play crucial roles in shaping ecological niches and have significant implications for health [1, 2], synthetic biology [3, 4], and environmental science [5, 6]. The advent of sequencing technologies has enabled researchers to amass vast microbiome datasets, significantly expanding our understanding of these complex systems [7]. Currently, hundreds of thousands of microbiome samples and their sequencing data have been accumulated and deposited in public databases [8, 9]. For example, as of 2023, EBI MGnify, a leading platform for microbiome data analysis and archiving, has cataloged 343,695 distinct samples from 4,601 studies across various biomes, including environmental, engineered, and host‐associated microbiomes [8]. While these extensive collections of microbial community samples represent a valuable resource, they also present challenges in integrating large‐scale microbiome data and extracting complex, multifaceted patterns within microbial communities, which is essential for advancing our understanding of their subtle evolutionary and ecological dynamics [10]. However, data heterogeneity, including insufficient data standards and a lack of interoperability across datasets, as well as profound batch effects among studies, limits the ability of traditional meta‐analytical methods in capturing shared insights across studies [11, 12, 13].

A promising approach for overcoming these limitations is the use of foundation models. These models are pretrained on large‐scale datasets, enabling them to generate a broad range of outputs. Recently, several studies have focused on developing a foundation model to improve the understanding of microbial sequence data [14, 15, 16]. These methods derive foundation models by training on vast, diverse datasets, capturing broad patterns that represent generalized biological relationships. For example, gLM, a genomic language model pretrained on millions of metagenomic scaffolds, demonstrated impressive zero‐shot performance in downstream tasks [16].

As the foundation model learned the shared knowledge embedded in a large‐scale dataset, it can be transferred to specific downstream tasks with the specific characteristics and nuances by transfer learning [17, 18]. This approach is often more effective than training models from scratch, as it leverages previously acquired knowledge, leading to improved performance and reduced training time [19, 20, 21]. By adjusting the learned representations to fit the context of the new dataset, transfer learning has shown potential for addressing the complexities of microbiome data integration and analysis [22, 23, 24].

However, these methods face inherent limitations due to their reliance on supervised learning strategies, which can introduce label bias during pre‐training. Consequently, different downstream tasks often necessitate the adoption of distinct pretrained models. Inappropriate pretrained model selection can lead to either negligible performance gains or even performance degradation in the contextualized model [23]. Moreover, inaccurately annotated microbiome samples, such as those labeled “Mixed biome” in MGnify, can distort the training process, leading to misinterpretations and ultimately hindering model performance.

To address these limitations, self‐supervised learning offers a compelling alternative. Unlike supervised methods, self‐supervised learning does not rely on labeled data and instead allows models to uncover underlying patterns in large datasets [25]. This approach circumvents the need for high‐quality labeled datasets and has shown significant promise in natural language processing (NLP), where large language models (LLMs) have evolved from simple pattern recognition to tackling more sophisticated tasks such as reasoning and content generation [26]. A key driver of these capabilities is the self‐attention mechanism [27], a core component of LLMs, which enables models to focus on relevant parts of high‐dimensional input data, capturing contextual relationships within text. By fine‐tuning these models on specific tasks, the knowledge gained during extensive pretraining on large corpora can be effectively transferred to new applications. This approach has led to significant performance improvements across a wide range of NLP tasks [28].

Recent advancements in the application of LLMs have shown promise in the analysis of biological tabular data [29, 30, 31, 32]. Building on their success in processing textual data, these models have been adapted to handle the structured, high‐dimensional nature of biological datasets. For example, scBERT employs a transformer‐based architecture to analyze gene expression data, enhancing tasks such as cell type classification and gene expression imputation [29]. Geneformer leverages pretrained LLMs to identify gene network regulatory elements within biological data [30]. Similarly, scGPT, inspired by generative pretraining, provides insights into cellular heterogeneity and simulates biological states under various conditions [31]. Lastly, scFoundation integrates foundation LLM techniques to create a versatile framework for diverse biological tasks, including cell type annotation, trajectory inference, and differential expression analysis [32]. These models underscore the potential of LLMs to revolutionize the analysis of biological tabular data, offering more accurate and comprehensive insights into complex biological systems.

However, these existing LLMs cannot be directly applied to microbiome abundance data due to a fundamental mismatch in vocabulary and learned representations. Single‐cell LLMs rely on gene‐centric vocabularies and embeddings, where tokens correspond to conserved genes with stable biological meanings. In contrast, microbiome data are organized around microbial taxa whose identities, resolutions, and functional relevance vary substantially across environments and studies. As a result, the biological knowledge encoded in pretrained single‐cell models is not transferable to the microbiome domain, motivating the development of a microbiome‐specific foundation model.

In this study, we introduce the Microbial General Model (MGM), a context‐aware, attention‐based foundation model specifically designed for microbiome analysis. MGM employs multi‐layer transformer blocks and is pretrained on over 260,000 microbiome samples from diverse biomes to capture generalizable microbial composition patterns. Through transfer learning, MGM replaces its language modeling head with task‐specific heads, enabling fine‐tuning on limited data for various applications. In benchmark evaluations, MGM outperformed traditional machine learning approaches and recent state‐of‐the‐art deep learning methods in microbiome analysis across multiple tasks. For example, in a cross‐regional disease diagnosis task, MGM generalized effectively across diverse cohorts, overcoming intercontinental variations in microbial community structures. In a longitudinal infant cohort, MGM distinguished between delivery modes, identifying developmental distinctions and keystone species. In tumor microbiome analyses, MGM uncovered potential therapeutic targets through in silico perturbation experiments. Additionally, we designed a prompt‐guided pipeline to generate realistic microbiome abundance profiles from prompts, facilitating controlled in silico experiments and simulations. Building on these results, we anticipate that foundation models like MGM will significantly enhance microbiome research and provide a powerful platform for future discoveries.

## 2 Results

### 2.1 Microcorpus‐260K and MGM Architecture

**FIGURE 1:**
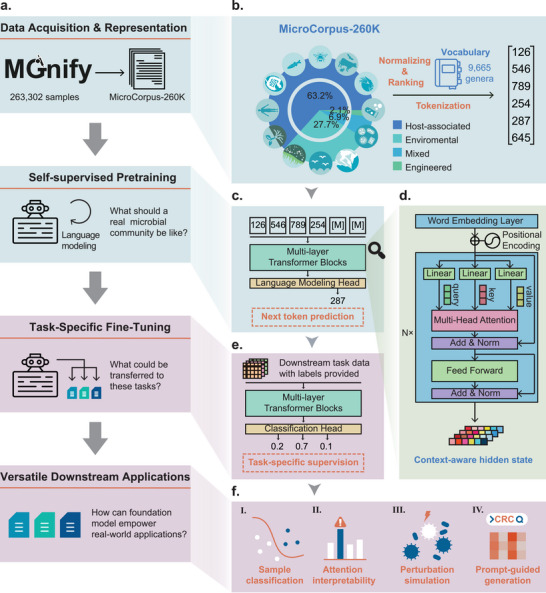
MGM architecture and transfer learning strategy. (a) High level workflow for MGM. (b) Construction process of Microcorpus‐260K.(c) MGM was pretrained using causal language modeling approach. (d) Model details in multi‐layer transformer blocks. (e) Adaptation of MGM to different downstream tasks using transfer learning methods. (f) Examples of downstream tasks: (I) Batch integration based on contextualized sample embedding. (II) Keystone species discovery based on contextualized attention weights. (III) Accurate predictions based on transfer learning. IV. In silico perturbation analysis.

MGM is a foundation model pretrained on large‐scale microbiome community samples from various biomes. An overview of the MGM framework is provided in Figure 1a, which illustrates the end‐to‐end workflow including microbiome data acquisition, context‐aware pretraining, task‐specific fine‐tuning, and downstream applications such as classification, prediction, and generation. We first assembled Microcorpus‐260K, a comprehensive dataset containing microbiome samples from the MGnify database up to June 2023 (Figure 1b). After filtering out low‐quality or incomplete data, 263,302 samples were retained for pretraining (Methods). From these samples, we generated a vocabulary comprising 9,665 distinct genera. The genera were normalized and ranked based on their relative abundance in each sample and then transformed into discrete input representations. This encoding strategy preserved the relative magnitudes of abundances among different genera and excludes the influence of extreme values. Given the fixed input length required by the transformer model, we selected 512 as the input length. This choice was made to ensure that 99.99% of the samples were adequately covered without truncation (Figure S1a). We further analyzed the genus frequency distribution across biomes, revealing, for example, that *Blautia* is prevalent in engineered and host‐associated samples but rarely observed in environmental ones (Figure S1c). Following the preparation of the dataset, we developed MGM using a multi‐layer transformer architecture (Figure 1d), designed to effectively capture the patterns and structures present within the large‐scale microbial community data.

### 2.2 Language Modeling Enables Generalizable Patterns Capture

We pretrained MGM using a causal language modeling approach on the Microcorpus‐260K dataset. During this process, the model progressively learned to predict the next genera based on existing microbial composition within the sample (Figure 1c). Unlike transformer encoder models such as BERT, this autoregressive pretraining strategy enables MGM to capture not only contextual dependencies but also community assembly patterns among genera within a sample. By leveraging the self‐attention mechanism, MGM captures high‐dimensional global representations of microbial community information (Figure 1d). For downstream tasks, MGM employs a transfer learning strategy, where the language modeling head is replaced by task‐specific heads (e.g., a sequence classification head) and the model is fine‐tuned on limited data (Figure 1e). This capacity makes it a versatile tool for a range of microbiome analyses, including batch integration, keystone species discovery, and microbial community classification. Additionally, MGM can predict the effects of perturbation by modifying the rank of certain microorganisms and generating realistic microbiome abundance profiles from prompts (Figure 1f).

**FIGURE 2:**
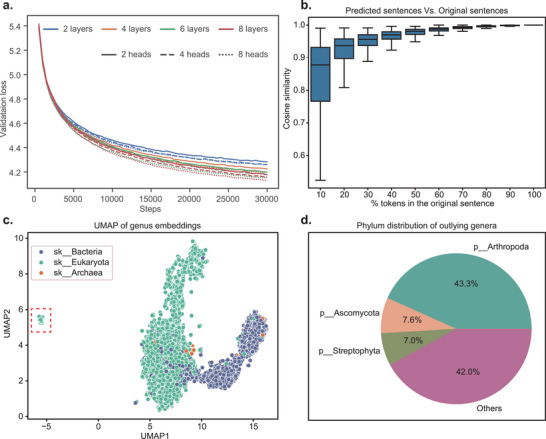
Hyperparameter search and pre‐training evaluation. (a) Grid search result of layers and heads of MGM. (b) Similarity between generated sentences and the original sentences. (c) UMAP visualization of the 9,665 genus embeddings extracted from MGM's word embedding layer. (d) Phylum‐level distribution of the 157 genera identified as outliers in the UMAP plot. Only the top 3 phylum have the most genera are labeled.

To optimize model performance, we conducted a grid search on hyperparameters and selected a structure comprising 8 layers and 8 attention heads (Figures 2a; S1a). To evaluate the effectiveness of the pretraining process, we randomly selected 1,000 samples from the validation set. For each sample, the pretrained MGM model predicted microbial compositions (referred to as “sentences”) based on a proportion of genera (referred to as “tokens”). We then compared the cosine similarity between the embeddings of the predicted and original compositions. Remarkably, even when only 20% of the original genera were provided, the cosine similarity between the predicted and original embeddings exceeded 0.9 (Figure 2b). These results highlighted MGM's strong ability to capture and generalize microbiome patterns, supporting its utility in diverse downstream microbiome analysis tasks.

We further explored whether the pretraining process captured taxonomic differences between genera, despite the absence of explicit phylogenetic information in our encoding strategy. We extracted embeddings for the 9,665 genera from the word embedding layer and found that genera from *Bacteria* and *Eukaryota* formed two diffuse clusters in the embedding space (Figure 2c). Additionally, we identified an outlier cluster comprising 157 genera, 43.3% of which belonged to *Arthropoda* (68 of 157, Figure 2d). Given that *Arthropoda* is not typically considered a core component in microbiome studies, this finding suggested that MGM is capable of detecting taxonomic distinctions, even without explicit phylogenetic encoding.

### 2.3 Microbial Community Classification and Batch Integration

We evaluated MGM through a comprehensive benchmark using a microbial community classification task on our Microcorpus‐260K dataset. This task represents a fundamental aspect of microbiome analysis, with applications such as microbial source tracking [33] and noninvasive diagnostics [34], both requiring high accuracy and strong generalizability.

We fine‐tuned MGM using cross‐entropy loss to predict biome names and lineages from MGnify and performed fivefold cross‐validation across hierarchical biome layers. As a baseline, we selected Random Forest (RF) due to its strong and stable performance in microbiome classification tasks. We further compared MGM with two state‐of‐the‐art deep learning models specifically designed for microbiome data: the ontology‐aware neural network EXPERT [23] and the phylogeny‐guided DeepPhylo [35], as well as the classical source tracking method FEAST [36]. To evaluate the value of self‐supervised pretraining, we also included a version of MGM without pretraining.

**FIGURE 3:**
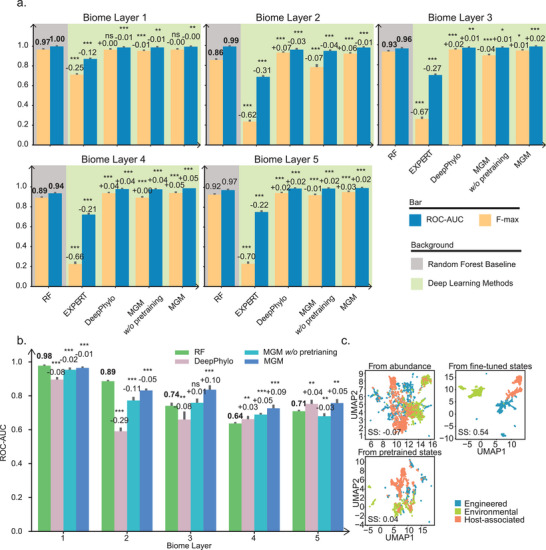
MGM Enhanced Source Tracking of Microbial Communities. (a) Barplot evaluation of the six methods using fivefold cross‐validation on each layer of biome lineage, with blue representing ROC‐AUC, yellow representing F‐max, and grey background representing Random Forest Baseline. (b) ROC‐AUC performance of each method on newly introduced data in MGnify. (c) UMAP visualization of 3,000 randomly selected samples based on microbial relative abundances, pretrained MGM embeddings, and fine‐tuned MGM embeddings, colored by the layer 1 biome. w/o, without. SS, silhouette score. Asterisks representing the significance of Wilcoxon test with false discovery rate (FDR)‐corrected: ^***^ *p* < 0.005, ^**^ *p* < 0.01, ^*^ *p* < 0.05, ns: not significant. Positive and negative bar values represent the mean performance difference relative to the Random Forest baseline (bolded values).

Our results show that fine‐tuned MGM achieved an average ROC‐AUC of 0.99, outperforming all competing methods and exhibiting a substantial mean performance gain relative to the RF baseline (Figure 3a). DeepPhylo and un‐pretrained MGM both reached an average ROC‐AUC of 0.97, comparable to RF, indicating that pretraining is critical to achieving state‐of‐the‐art performance. Notably, EXPERT achieved a considerably lower average ROC‐AUC of 0.61, failing to outperform even the traditional RF model. The FEAST method, based on expectation‐maximization, achieved an ROC‐AUC of 0.68 at the first biome layer but was excluded from deeper‐layer evaluation due to its prohibitively high computational cost on large‐scale datasets (Figure S1e). We also assessed additional classical machine learning models, including K‐Nearest Neighbors (KNN) and Logistic Regression (LR), whose performances remained below the RF baseline. This further justifies our selection of RF as the primary traditional comparison (Figure S1d). Notably, inference with MGM is highly efficient: on a single NVIDIA A100 GPU, the model can process 100,000 samples in under 25 s, demonstrating its scalability for large‐scale microbiome analyses (Figure S1e).

To evaluate generalization, we applied the pretrained MGM model to 43,528 newly released MGnify samples (post‐June 2023) without any fine‐tuning. While RF performed slightly better at coarse biome levels (Layers 1 and 2), MGM significantly outperformed RF at deeper, more complex levels (Layers 3–5) (Figure 3b). Additionally, both fine‐tuned and pretrained MGM embeddings achieved better clustering performance than raw abundance profiles (Figure 3c), demonstrating that MGM learns transferable representations that generalize across diverse datasets. These findings highlight the critical role of pretraining in microbiome foundation models and establish MGM as a robust and extensible framework that surpasses both traditional and deep learning baselines in accuracy and generalization.

### 2.4 Overcoming Cross‐Regional Limitation

Accurate microbiome‐based diagnosis across geographic regions remains challenging due to variability in microbial compositions driven by environmental, dietary, and host genetic differences. Models trained on region‐specific data often fail to generalize, especially when external datasets differ significantly in distribution. A robust diagnostic framework must capture generalizable microbial features while remaining adaptable to local variation.

**FIGURE 4:**
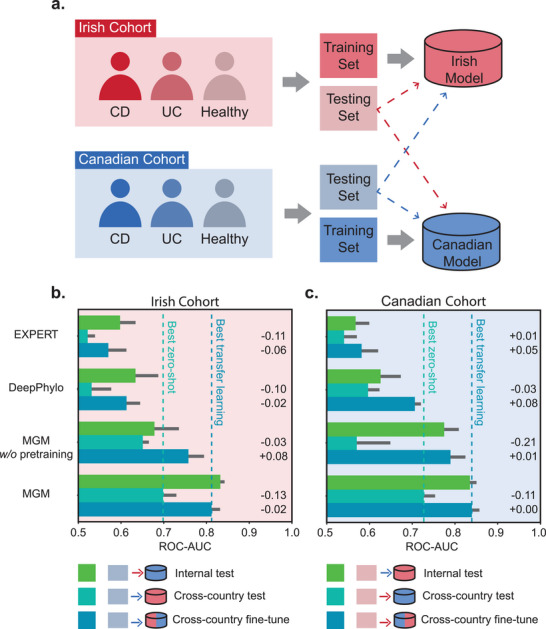
Result of disease diagnosis across intercontinental regions. (a) Benchmark experiment setting for cross‐regional disease diagnosis. (b) The performance of models trained on the Irish cohort in cross‐regional diagnosis. (c) The performance of models trained on the Canadian cohort in cross‐regional diagnosis. w/o, without. Positive and negative bar values represent the mean performance difference relative to the internal test.

To assess MGM's robustness to regional variation in clinical diagnosis, we evaluated its performance on an inflammatory bowel disease (IBD) cohort [37] comprising samples from Ireland and Canada. We found that *Lachnoclostridium* was ranked lower in Crohn's disease samples compared to healthy controls and also ranked lower in Canadian samples compared to Irish samples (Figure S2). Based on this, we designed a cross‐regional evaluation in which models were trained on one region (source) and tested directly on another (target) to assess zero‐shot cross‐regional performance. Subsequently, we performed transfer learning using a subset of samples from the target region to adapt the models (Figure 4a).

We compared MGM with EXPERT, DeepPhylo, and an un‐pretrained version of MGM. As expected, all models performed worse in the zero‐shot setting than in internal testing, but performance improved after transfer learning. Notably, the zero‐shot cross‐regional performance of MGM exceeded the transfer learning performance of both EXPERT and DeepPhylo, demonstrating MGM's ability to achieve accurate diagnosis even without access to target‐region data (Figure 4b,c).

These results demonstrate that MGM effectively models spatial microbial dynamics and that a foundation diagnostic model trained on heterogeneous data can mitigate regional biases, offering a scalable and generalizable solution for microbiome‐based disease prediction across populations.

### 2.5 Infant Development Monitoring and Keystone Genus Discovery

Next, we fine‐tuned our model on a longitudinal infant gut microbiome dataset from Roswall et al. [38] to track the dynamic progression of gut microbial communities across developmental stages and delivery modes. During infancy, the gut microbiome undergoes rapid shifts before stabilizing in childhood, with delivery mode playing a crucial role in shaping early microbial colonization, which can influence long‐term health outcomes [39].

**FIGURE 5:**
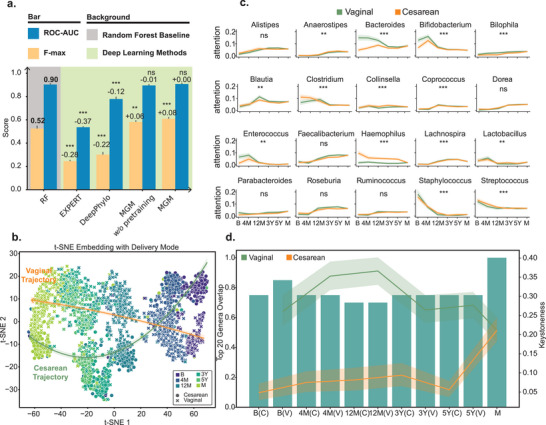
MGM Enhanced age prediction and keystone genus discovery. (a) Barplot evaluation of MGM model using fivefold cross‐validation, with blue representing ROC‐AUC, yellow representing F‐max, and grey background representing Random Forest Baseline. w/o, without. Asterisks representing the significance of Wilcoxon test with false discovery rate (FDR)‐corrected: ^***^ *p* < 0.005, ^**^ *p* < 0.01, ^*^ *p* < 0.05, ns: not significant. Positive and negative bar values represent the mean performance difference relative to the Random Forest baseline (bolded values). (b) Line plot showing the attention weights of the top 20 genera across layers and heads. Asterisks representing the significance of Kolmogorov–Smirnov (KS) test: ^***^ *p* < 0.005, ^**^ *p* < 0.01, ^*^ *p* < 0.05, ns: not significant. (c) t‐SNE visualization of sample embedding across development stage, shaped by different delivery mode. (d) Line plot showing the keystoneness scores of genera present in ≥30% of samples within each group. The accompanying green bar plot indicates the overlap between the top 20 genera ranked by attention weights and those ranked by keystoneness. B: birth, 4M: 4 months, 12M: 12 months, 3Y: 3 years, 5Y: 5 years, C: cesarean, V: vaginal, M: mother.

We compared the performance of MGM with RF, EXPERT, DeepPhylo, and an un‐pretrained version of MGM on the task of predicting infant developmental stages. MGM accurately predicted both developmental stage and delivery mode, outperforming RF and other deep learning‐based methods. Specifically, MGM achieved an AUROC comparable to that of the RF baseline while exhibiting a substantially higher F‐max (MGM: AUROC = 0.90, F‐max = 0.60; RF: AUROC = 0.90, F‐max = 0.52; Figure 5a), corresponding to a relative improvement of approximately 15% in F‐max. Although the AUROC difference between MGM and RF was not statistically significant, the increased F‐max reflects a more favorable balance between precision and recall at the optimal operating point, implying that MGM yields fewer false‐positive associations while maintaining sensitivity to true microbial signals. In contrast, other deep learning‐based methods, including EXPERT and DeepPhylo, consistently underperformed relative to the RF baseline (EXPERT: AUROC = 0.54, F‐max = 0.24; DeepPhylo: AUROC = 0.78, F‐max = 0.30; Figure 5a), highlighting the advantage of self‐supervised pretraining and attention‐based architectures. Furthermore, t‐SNE visualization of sample embeddings revealed clear temporal structuring of microbial communities. Microbiomes from cesarean and vaginal deliveries exhibited stage‐specific patterns, and as infants aged, their microbiota increasingly resembled that of their mothers, supporting the hypothesis that the infant gut microbiome gradually matures toward an adult‐like composition (Figure 5b).

We then examined the attention weights from 64 attention heads across developmental stages. Several genera, including *Dorea*, *Faecalibacterium*, and *Ruminococcus* (KS test, *p* = 0.13, 0.19, and 0.22), demonstrated similar attention trends across delivery modes, consistent with their known associations with infant age [40]. However, most genera demonstrated distinct attention patterns. Notably, *Bacteroides* (KS test, *p =* 5.87E‐5), a keystone taxon of the human gut microbiota [41], and *Bifidobacterium* (KS test, *p =* 1.53E‐12), a common probiotic [42], received higher attention weights in vaginal deliveries during early stages. In contrast, *Haemophilus* (KS test, *p =* 4.88E‐90), a well‐known human pathogen, had consistently higher attention weights in cesarean deliveries throughout the entire developmental process (Figure 5c).

We further employed a leave‐one‐genus‐out deletion approach to identify genera whose removal would have a deleterious effect, defined here as a substantial perturbation in the sample embedding caused by removing a genus from the rank value encoding. In this analysis, one genus was removed from the rank value encoding at a time, and the impact on the embeddings of the remaining genera was quantified by similarity. The deleterious pattern of two delivery modes is consistent with the results of the attention weights analysis that probiotic has higher deleterious effects on infants delivered vaginally. Keystone taxa such as *Bacteroides*, *Roseburia*, and *Faecalibacterium* exhibited the higher deleterious effects (Figure S3a). Based on these findings, we hypothesize that genera with high attention weights and deleterious effects possess strong keystone attributes.

We quantified the keystone attributes of genera using the DKI framework [43], a deep‐learning model designed to assess community‐specific keystoneness by conducting a thought experiment on species removal. We found that genera with high attention weight had a large overlap with genera with high keystoneness, and these genera were present in a large proportion of samples. Interestingly, infants delivered vaginally exhibited higher overall keystoneness compared to those delivered by cesarean section, with the mother's keystoneness was intermediate between the two delivery modes. (Figures 5d; S3b). We also performed a comparative analysis between attention weights and SHAP (Shapley Additive exPlanations) [44] values for genus‐level features across developmental stages, showing substantial overlap in the top 20 genera (Figure S4a).

In summary, our attention‐based model effectively identified keystone taxa and captured the developmental trajectory of the infant gut microbiome. These results demonstrated MGM's ability to model temporal microbial dynamics, offering a powerful framework for studying microbial influences across the human lifespan.

### 2.6 Potential Cancer Treatment Target Identification

We next explored MGM's capability to differentiate cancer types and identify tumor‐specific biomarkers, a crucial step toward precision oncology. Recent studies have increasingly highlighted the presence of microbial signals within tumor tissues, suggesting that the tumor microbiome could be a valuable target for cancer research and treatment [45, 46, 47]. However, the research on biomarkers across different tumor tissues may not yet be sufficiently comprehensive [48]. This underscores the need for more robust models that can effectively differentiate cancers and identify specific microbial biomarkers, which could be crucial for developing targeted therapies.

**FIGURE 6:**
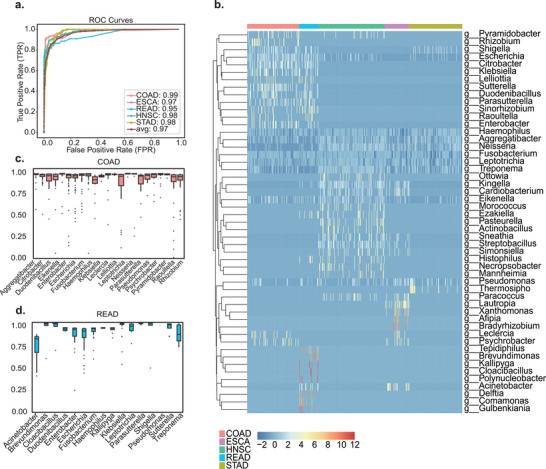
MGM Enhanced Cancer Diagnosis and Biomarker Identification. (a) ROC performance of MGM in distinguishing between five types of cancer. The x‐axis represents the False Positive Rate (FPR), while the y‐axis indicates the True Positive Rate (TPR). The macro‐average ROC value reaches 0.97, with individual ROC values as follows: COAD (0.99), ESCA (0.97), HNSC (0.98), READ (0.98), and STAD (0.97). (b) Distribution of the top 50 species identified by attention across the five types of cancer. The x‐axis represents different cancer types (COAD, ESCA, HNSC, READ, STAD), and the y‐axis lists the microbial genera. The color intensity in each cell corresponds to the abundance level, with the scale ranging from −2 to 12, where positive values indicate a higher relative abundance. (c,d) Cosine similarity of embedding vectors before and after the removal of a specific genus in COAD and READ. The x‐axis shows the microbial genera removed, and the y‐axis represents the cosine similarity score, ranging from 0 to 1.

To this end, we fine‐tuned our model using five types of gastrointestinal tumors obtained from The Cancer Microbiome Atlas (TCMA) database [49]. Our model achieved an exceptional macro‐ROC of 0.97, demonstrating unparalleled diagnostic accuracy across multiple cancer types. (Figure 6a). Specifically, ROC values for COAD, ESCA, HNSC, READ, and STAD were 0.99, 0.97, 0.98, 0.98, and 0.97, respectively, highlighting MGM's robust generalization across diverse tumor microenvironments.

To identify the potential therapeutic targets, we employed the ‘leave‐one‐genus‐out’ approach to assess the impact of each genus's absence on the sample embeddings. We extracted the top 50 biomarkers ranked by MGM‐calculated attention scores, and the heatmap (Figure 6b) illustrated their distribution across the five cancer tissues. Our findings suggested that some genera exhibited significant abundance differences in particular types of cancer. For instance, *Escherichia* and *Enterobacter* had a significant detrimental impact on COAD and READ samples. These genera have been previously associated with gastrointestinal tumors [49, 50, 51]. In COAD samples, the abundance of *Escherichia* was observed to be 7.25 times higher than in non‐COAD samples. Conversely, genera like *Acinetobacter* may act as key biomarkers distinguishing COAD and READ tissues. *Streptobacillus*, another significant genus, displayed an elevated presence in HNSC, with a relative abundance increase of 5.26 times compared to other types, indicating its importance in distinguishing HNSC. Boxplots (Figure 6c,d) showed the cosine similarity of the embedding vectors before and after the removal of a specific genus. Notably, when *Acinetobacter* was removed, the cosine similarity in the READ samples significantly decreased, underscoring the critical role this genus plays in shaping the microbial composition of these samples. We validated the in silico perturbation results using SHAP on the TCMA dataset. In each cancer type, the top 5 genera identified as having the most deleterious effects showed substantial overlap with the top SHAP‐ranked genera, supporting the biological relevance of the perturbation framework (Figure S4b). These findings not only validated the effectiveness of the MGM model in identifying cancer‐associated microbial biomarkers but also offered new possibilities for targeted cancer diagnosis and treatment.

Collectively, our model demonstrated high diagnostic accuracy and robust performance across multiple cancer types, underscoring the crucial role of the tumor microbiome in oncogenesis. These advancements revealed MGM's potential for more precise and targeted cancer therapies, highlighting the potential of integrating tumor microbiome analysis into clinical practice.

### 2.7 Prompt‐Guided Disease‐Specific Microbiome Generation and Evaluation via a Microbiome Turing Test

**FIGURE 7:**
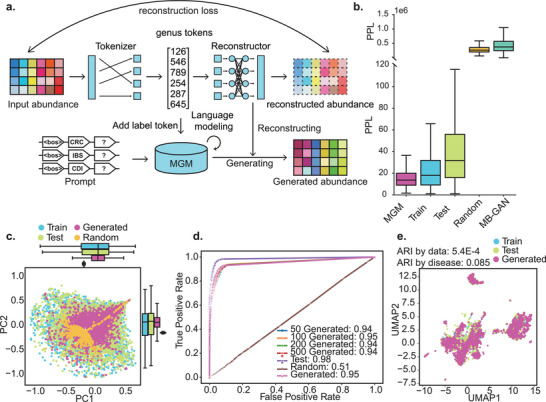
Evaluation of the prompt‐guided MGM generative model. (a) MGM generative model pipeline. (b) Perplexity of different datasets. Perplexity in language models measures the degree to which the model considers a sentence to be realistic. (c) Beta diversity analysis of the reconstructed model, based on Bray‐Curtis distance. (d) ROC‐AUC curves of the classifier for gradient‐generated data. (e) UMAP dimensionality reduction plots of embeddings for three data types (train, test, generated). The ARI indices represent clustering based on data type and disease type, with clustering labels obtained through a Gaussian Mixture Model.

To demonstrate the generative capabilities of MGM, we implemented a prompt‐guided pipeline for disease‐specific microbiome synthesis. Given that rank‐based encoding discards absolute abundance information, a reconstructor network was trained to convert generated rank sequences back into abundance profiles. Label tokens corresponding to disease categories were appended after the beginning‐of‐sequence token to guide conditional generation. Fine‐tuning was performed via next‐token prediction, allowing disease prompts to steer the synthesis of sample‐like sequences, which were subsequently reconstructed into abundance tables (Figure 7a, Methods).

Using 6,004 gut microbiota samples covering 17 diseases from the GMrepo database, we trained and evaluated the model via hierarchical sampling into equal training and testing sets. Generated samples exhibited the lowest perplexity among all data types, indicating strong sequence coherence (Figure 7b). Bray‐Curtis beta diversity analysis showed that synthetic samples closely matched the distribution of real samples (Figure 7c). An RF classification model trained on real data achieved a ROC‐AUC of 0.98 on held‐out real samples and 0.95 on generated ones, suggesting strong retention of disease‐specific signatures. Classifier performance remained consistent when varying the number of generated samples per disease (50, 100, 200, 500), indicating robustness (Figure 7d). UMAP projection of sequence embeddings revealed that generated samples clustered more distinctly by disease (ARI = 0.085) than by source type (train/test/generated; ARI = 5.4E‐4), further supporting the model's ability to capture biologically meaningful structure (Figure 7e).

To systematically assess MGM's generative quality, we developed a Microbiome Turing Test (Table 1) comprising two phases: statistical fidelity and biological relevance. In the first phase, statistical evaluation showed strong alignment between generated and real profiles. The Mean Absolute Error between relative abundances was 0.00013 ± 1e‐05. Cosine similarity reached 0.52 ± 0.069, and entropy‐based sparsity measures were closely matched (real: ∼2.4; generated: 2.4 ± 0.24), indicating similar abundance distributions. Spearman correlation between taxa abundances was 0.51 ± 0.05, reflecting preserved intra‐community relationships.

**TABLE 1:** Microbiome Turing Test of Different Methods. This table summarizes the performance metrics of three different methods: MGM, MB‐GAN, and Random, along with a baseline for comparison. Each metric is reported with its mean value and 95% confidence interval where applicable.

Metrics	True	MGM	MB‐GAN	Random
Phase 1				
Cosine Similarity	—	0.52 ± 0.069	0.046 ± 0.0077	0.05 ± 0.0084
MAE	—	0.00013 ± 1e‐05	0.00011 ± 8.3e‐07	0.00011 ± 6e‐07
Alpha diversity	2.8 ± 0.33	3.4 ± 0.3	1.5 ± 0.055	1.6 ± 0.026
Sparsity	1.9 ± 0.23	2.4 ± 0.24	1.1 ± 0.038	1.1 ± 0.018
Spearman	—	0.51 ± 0.05	0.0018 ± 0.00073	0.0015 ± 0.00063
Phase 2				
ROC‐AUC	—	0.95	0.49	0.5
Biomarkers Overlap (Top 500)	500	440	287	283
Network Density	0.18	0.30	0.10	0.10
Network average degree	281.16	**351.88**	959.98	959.98
Network modularity	0.22	**0.16**	0.13	0.13

Biological relevance was further validated through multiple downstream analyses. A random forest classifier trained on real data maintained high accuracy (ROC‐AUC = 0.95) when applied to synthetic samples. Conversely, classifiers trained on generated data identified key taxa highly consistent with those from real data, sharing 88% (440/500) of the top‐ranked features. Co‐occurrence network analysis revealed similar structural properties between real and generated microbiomes, with comparable network density (0.30), average degree (351.88), and modularity (0.16), confirming the preservation of community‐level organization.

Comparative evaluation against MB‐GAN [52], a GAN‐based microbiome generator, showed that MGM outperformed it on 8 of 10 Microbiome Turing Test metrics (Figure 7b and Table 1). In contrast to MB‐GAN, which requires retraining for each disease, MGM supports efficient, condition‐specific generation via prompt tokens within a unified framework. These results highlight MGM's capacity to generate high‐fidelity, biologically informative microbiome profiles, offering a versatile tool for synthetic data generation in microbiome research.

## 3 Discussion

In this study, we proposed MGM, the first foundation model designed for microbial community analysis, leveraging pretrained transformers on a diverse corpus of microbiome data. Through large‐scale self‐supervised pretraining, MGM departs from conventional pipelines that analyze microbiome datasets in isolation and instead learns generalizable, context‐aware representations of microbial community structure across cohorts, diseases, and environments. This general representation captures broad patterns and relationships across varied microbiome datasets, establishing MGM as a versatile tool in microbiome research.

MGM employs a contextualization approach that reframes microbiome analysis as a unified representation learning problem. Fine‐tuning MGM on task‐specific datasets enhances its ability to capture nuanced microbial relationships while retaining the robustness of it pretrained knowledge. Through systematic analysis, we demonstrate that a pretrained MGM backbone coupled with task‐specific heads consistently outperforms traditional statistical approaches and recent deep learning methods across diverse downstream tasks.

Benchmark evaluations underscore MGM's superior performance in microbial community classification tasks. In cross‐validation on the Microcorpus‐260K dataset, the fine‐tuned MGM achieved an average ROC‐AUC of 0.99, significantly outperforming traditional methods, including source tracking techniques, machine learning models, and recent state‐of‐the‐art deep learning methods. Its application to 43,528 additional samples from MGnify revealed exceptional performance in deeper, more complex analyses. Furthermore, MGM embeddings enabled seamless integration of microbiome data across different sources and batches, highlighting its utility in distinguishing microbial samples for tasks such as microbial source tracking.

Beyond classification, MGM captures the spatial and temporal dynamics of microbial communities. In cross‐regional intestinal disease datasets, MGM overcame regional limitations, achieving accurate diagnoses across intercontinental regions. When applied to a longitudinal infant dataset, the model effectively traced the development and maturation of the infant gut microbiome. Attention‐weight analyses identified key genera, including *Bacteroides* and *Bifidobacterium*, as keystone taxa in vaginal deliveries, while *Haemophilus* showed sustained dominance in cesarean deliveries, supporting their potential role in early‐life microbiome establishment.

MGM also demonstrated its robust generative capabilities for microbiome data generation. Through prompt‐guided generation, the model could produce realistic, biologically meaningful microbiome profiles conditioned on specific contexts, such as disease states. The Microbiome Turing Test, a comprehensive evaluation framework incorporating biological significance and data distribution metrics, validated the superiority of MGM over methods like MB‐GAN. This capability is particularly valuable for generating synthetic datasets, which can augment limited real‐world data, facilitate machine learning model development, and explore hypothetical microbiome states.

While MGM shows promise, several limitations should be acknowledged. First, the current framework relies on a rank value encoding method, which effectively mitigates the impact of extreme values and converts tabular data into a sequential representation. However, this encoding inevitably sacrifices absolute and relative abundance information. To overcome this issue, future work should explore more expressive encoding strategies such as hybrid encoding, which can integrate the robustness of rank‐based methods with the fidelity of abundance‐preserving features. Second, potential sampling bias and dataset representativeness poses an important limitation. A substantial proportion of the microbiome samples used for pretraining are derived from the MGnify resource, in which more than 60% of the entries correspond to host‐associated microbiomes (Figure 1b). This imbalance may bias the learned representations toward host‐related microbial patterns and limit the model's performance on environment‐associated microbiome tasks. Future work will therefore prioritize expanding both the training and evaluation sets with more diverse environment‐associated microbiomes, such as soil, marine, and engineered ecosystems, and systematically assessing the model's transferability across distinct ecological contexts. Finally, while the model shows great promise in identifying treatment targets and keystone genera, the current conclusions are primarily based on computational analyses. Incorporating wet‐lab experimental validation, such as microbial perturbation assays, will be an essential next step to substantiate the biological relevance and translational value of the model predictions.

In conclusion, MGM represents a profound advancement in microbiome research, offering a robust and adaptable tool for analyzing microbial communities. As a foundation model, MGM exceled in large‐scale microbial classification, leveraging vast pretrained datasets to capture fundamental patterns that span diverse microbial ecosystems. In its contextualized form, MGM could be fine‐tuned for specific downstream tasks, such as identifying clinically relevant microbial perturbations and uncovering nuanced microbial interactions. This dual capacity to model both general and task‐specific patterns underscored its broad applicability in microbiome science, including therapeutic interventions and diagnostic innovations. As a microbiome foundation model that unifies representation learning, transfer learning, perturbation analysis, and data synthesis within a single framework, MGM lays the groundwork for future innovations in microbiome science and personalized medicine.

## 4 Methods

### 4.1 Data Preprocessing

We assembled a comprehensive dataset, Microcorpus‐260K, which includes all samples from MGnify up to June 2023. Initial processing involved retaining genus‐level relative abundances and filtering out genera with relative abundances less than 0.01%. Samples were further filtered to retain only those with at least 10 genera with non‐negligible abundance, resulting in a final dataset of 263,302 samples and a vocabulary of 9,665 genera.

Each microbial community sample, denoted as {$x_{1},x_{2},x_{3},\text{…},x_{n}$}, consists of a set of microbial taxa with non‐zero relative abundance, where *x_i_* denotes the relative abundance of genus *i*. Genera with zero abundance are excluded from encoding. The vocabulary size *X* corresponds to the total number of unique genera in the dataset. To standardize abundances, each *x_i_* was normalized using the dataset‐wide mean μ_*j*_​ and standard deviation σ_*j*_​:

$$x_{i}^{\prime }=\frac{x_{i}^{\prime }-\mu_{j}}{\sigma_{j}}$$

Normalized abundances were then ranked within each sample, with the highest assigned rank 1. The rank *r_i_* ​ of genus *i* is defined as:

$$r_{i}=rank\left(x_{i}^{\prime }\right)$$

Ranks were then tokenized into discrete representations, mapping each rank *r_i_* ​ to a unique token t_i from the vocabulary $V=\{t_{1},t_{2},\text{…},t_{X}\}$. This transformation encodes microbial communities as sequences of tokens:

$$\left\{t_{r_{1}},t_{r_{2}},\text{…},t_{r_{n}}\right\}$$

This approach captures the relative structure of microbial communities while reducing dependence on absolute abundance values. We compared rank‐based encoding with CLR (centered log‐ratio)‐based encoding using a masked language model on the microbial community classification task. The CLR model achieved a lower AUROC (0.97 vs. 0.99), suggesting that rank‐based encoding better captures contextual patterns in microbiome data (Figure S5).

### 4.2 Model Architecture

We constructed the MGM model using eight layers of transformer blocks, with each block consisting of a self‐attention layer and a feed‐forward neural network layer. Given the fixed input length requirement of transformer models, we set the input length to 512 tokens, covering 99.99% of the samples. This length ensured that most samples could be processed without truncation, preserving the integrity of the data. Additional key hyperparameters were as follows: activation function, Gaussian Error Linear Unit (GELU); attention heads per layer, eight; feed forward size, 1024. The modeling framework was implemented in PyTorch, leveraging the Huggingface Transformers library for model configuration and training [53].

The self‐attention mechanism employed in each transformer layer follows the scaled dot‐product attention formula:

$$\mathrm{Attention}\left(Q,K,V\right)=\mathrm{softmax}\left(\frac{QK^{T}}{\sqrt{d_{k}}}\right)V$$

where *Q* (queries), *K* (keys), and *V* (values) are the linear projections of the input, and *d_k_* is the dimensionality of the keys. This formulation allows the model to capture contextual relationships between different tokens in the sequence, enabling more effective representation learning for downstream tasks.

### 4.3 Pretraining Procedure

Pretraining was conducted using the causal language modeling approach with a self‐attention mechanism to capture co‐occurrence patterns among tokens. For each sample, we appended a ‘bos’ token at the beginning and an ‘eos’ token at the end to denote the start and end of a sequence, respectively. We adopted causal language modeling (CLM) as the pretraining objective, which allows the model to sequentially process the entire microbial community sample and update every token during training. Compared to masked language modeling (MLM), which only partially utilizes the input per step, CLM enables more efficient context learning. Furthermore, CLM naturally supports prompt‐guided generation, which we leverage in downstream tasks such as microbial community synthesis. An MLM‐based variant of our model achieved an average AUROC of 0.92 on the classification task but still fell short of the performance achieved by the CLM‐based model (Figure S5).

Training was executed using Huggingface's Trainer API. Key hyperparameters included: learning rate, 1e‐3; batch size, 50; warmup steps, 1000; weight decay, 0.001; validation split, 10% of the data. Validation loss is calculated per 500 training steps, Early stopping based on validation loss with 3 patience.

### 4.4 Model Interpretability

We conducted an interpretability analysis by leveraging the attention weights extracted from the multi‐head, multi‐layer transformer. These attention weights were modified by replacing *V* with *V* ^0^, where *V* ^0^ represents one‐hot indicators for each position index. To consolidate the attention information across the model, we integrated the attention matrices by calculating an element‐wise average across all layers and attention heads. To identify the genera with the highest attention weights in a microbial community, we summed the attention weights across each column, obtaining the total attention weight from a single genus to all other genera in the community.

### 4.5 Sample Representation

Each sample is analogous to a ‘sentence’ composed of genera, and its representation is obtained by aggregating the learned genus‐level representations. In this study, we opted element‐wise mean pooling to get the sample representation from our pretrained model. For fine‐tuned model, as the last token (‘eos’ in this study) was used to do the sequence classification, we used its embeddings as the sample representation.

### 4.6 Downstream Fine‐Tuning

For downstream tasks, the pretrained MGM model is fine‐tuned by replacing the language modeling head with a task‐specific head. All downstream tasks in this study focused on microbial community classification. Fine‐tuning employed a sequence classification head, which utilized the final token (‘eos’ in this study) for classification. During task‐specific fine‐tuning, all layers of the pretrained MGM backbone were unfrozen and updated jointly with the task‐specific head, enabling full adaptation to downstream tasks. Fine‐tuning was executed using Huggingface's Trainer API. Key hyperparameters included, learning rate, 1e‐3, batch size, 50, warmup steps, 1000, weight decay, 0.001, and validation split, 10% of the data. For microbial classification task on MicroCorpus‐260K, For the microbial classification task on the MicroCorpus‐260K dataset, validation loss was calculated every 500 training steps, while for other downstream tasks, it was calculated per training epoch. Early stopping based on validation loss with 3 patience.

The evaluation of both the microbial classification task and the infant age prediction task was performed using a fivefold cross‐validation strategy. Training was conducted on 80% of the samples, with performance tested on the 20% held‐out samples, and this process was repeated across five folds. For the cross‐regional disease diagnosis task, 20% of the samples from each region were used as the test set using stratified sampling, while the remaining 80% were used either to train the diagnostic model or fine‐tune a model trained on another region. Notably, the fine‐tuning applications were trained on classification objectives distinct from the causal language modeling objective, making the inclusion of task‐specific data in the pretraining corpus irrelevant to classification predictions.

### 4.7 Prompt‐Guided Generation Process

To adapt the model for prompt‐guided generation, we expanded the model's vocabulary by introducing a new token immediately following the ‘<bos>’ (beginning‐of‐sequence) token, designated as the label token. Let the extended vocabulary be denoted as $V^{\prime }=\left\{t_{bos},\;t_{eos},t_{1},t_{2},\text{…},t_{X},t_{label}\right\}$, where ​ *t_bos_* corresponds to the ‘<bos>’ token, *t_eos_* corresponds to the ‘<eos>’ token, $t_{1},t_{2},\text{…},t_{X}$ are the tokens representing the ranks of genera, and *t_label_* ​ is the new token added to indicate the sample identity. Each sample in the microbiome dataset is then associated with a unique label token $t_{label}^{i}$​, where *i* corresponds to the specific microbiome sample. For each microbiome sample $S_{i}=\left\{x_{1},x_{2},x_{3},\text{…},x_{n}\right\}$, we assign it a label token $t_{\mathrm{label}}^{i}\in V^{\prime }$ to represent the sample identity, so that the model can generate a sequence specific to that sample.

This vocabulary expansion ensures that the model can not only generate microbiome sequences but also tailor the sequences to reflect the characteristics of a specific sample. Formally, the input to the model is now a sequence of tokens $\left\{t_{bos},t_{label}^{i},t_{r1},t_{r2},\text{…},t_{rn},\;t_{eos\;}\right\}$, where $t_{label}^{i}$​ denotes the label token for the sample *i* and $t_{r1},t_{r2},\text{…},t_{rn}$ are the tokens representing the ranks of microbial genera in the sample.

Once this vocabulary expansion is made, the model is fine‐tuned using next‐token prediction. The training objective is to predict the next token in the sequence given the previous tokens. Let the current sequence of tokens be $\left\{t_{1},t_{2},\text{…},t_{k}\right\}$, and the model's goal is to predict the next token *t*_*k* + 1_. The loss function used for training is the negative log‐likelihood of predicting the correct token *t*_*k* + 1_​, given the sequence of previously generated tokens:

$$L\left(\theta \right)=-\sum_{k=1}^{n}\log P\left(t_{k+1}|t_{1},t_{2},\text{…},t_{k};\theta \right)$$

Here, θ represents the model parameters, and $P(t_{k+1}|t_{1},t_{2},\text{…},t_{k};\theta )$ is the probability predicted by the model for the next token *t*_*k* + 1_​ conditioned on the previous tokens. This is learned by minimizing the cross‐entropy loss over the entire sequence. During the generation phase, the model is provided with the label token *t*_*k* + 1_ as the prompt, which signals the model to generate a sequence corresponding to the sample *i*. The model then generates a sequence of tokens $\left\{t_{bos},t_{label}^{i},t_{r1},t_{r2},\text{…},t_{rk},t_{eos}\right\}$, where each *t_rk_* ​ represents the rank of the *k^th^* genus in the sample.

### 4.8 Reconstructor Architecture

Once the model generates the sentence‐like sequence, it is necessary to convert these sequences back into relative abundance values. To achieve this, we utilized the reconstructor network that was trained in parallel with the main model. The reconstructor's task is to take the generated rank sequence and map it back to the original relative abundance scale. This step ensures that the final generated microbiome sequences are not only in the correct rank order but also reflect the original quantitative properties of the microbiome samples.

The reconstructor network was trained using a dataset of paired rank‐encoded and abundance‐encoded samples, learning to approximate the mapping from sentence‐like representation back to relative abundance values. By using this approach, we preserve the diversity and abundance distributions of the microbiome, enabling the generation of realistic, contextually accurate microbiome data.

The reconstructor is a deep‐learning model used to reconstruct microbial abundance. The ranked corpus after tokenizing was first encoded to a vector $X\;\in \;{\left[0,\;2\right]}^{N}$, where

*X_i_* = 0 if species *i* is absent from this sample and *X_i_* = *PE*(*i*)  + 1 if it is present. Here, *PE*(*i*) is the position embedding from the Transformer with *d_model_* = 1. The microbial composition of this sample is represented by a vector $Y\;\in \;{[0,1)}^{N}$, where *Y_i_* is the relative abundance of species *i* in this sample. This deep‐learning model (ReconstructorNet) was trained to learn the map from *X* to *Y*. This model is a three‐layer neural network with layer sizes *N* × 2*N* × *N* × *N*, using ReLU activation in the first two layers and Softmax in the final layer, optimized using Adam. The last two layers also employ residual connections to improve training stability and performance.

Pytorch‐Lightning was used to build and train the model. Key hyperparameters included: Learning rate: 2e‐4, Batch size: 64, Validation split: 20% of the data, Early stopping based on validation loss with 10 patience.

### 4.9 Microbiome Turing Test

In the Microbiome Turing Test framework, we computed five first‐order metrics and five second‐order metrics. For the first‐order metrics, cosine similarity, mean absolute error (MAE), Spearman correlation coefficient, alpha diversity, and sparsity were calculated by comparing the generated data with the real data for each disease individually, and then averaging the results across 17 diseases to obtain the evaluation metric. Specifically, cosine similarity was computed using the cosine_similarity function from the sklearn metrics pairwise module, while MAE was calculated by taking the mean absolute differences between the real and generated data for each sample. Spearman correlation coefficients were determined using the spearmanr function from scipy.stats module. Alpha diversity was assessed using Shannon entropy and Simpson index, calculated with the alpha_diversity function from the skbio.diversity module. Sparsity was evaluated using entropy, and the distributions of these entropy values were compared using the Wilcoxon signed‐rank test, implemented in scipy.stats module.

The second‐order metrics focused on the biological significance of the data. For ROC‐AUC, a Random Forest classifier trained on the real data was used to test the generated data from each model. The overlap of biomarkers was assessed by identifying the top 500 most important features in Random Forest classifiers trained on each dataset and then calculating the number of overlapping biomarkers between the real and generated data. Finally, we constructed Pearson correlation networks from the real and generated data using the networkx library. We evaluated network properties included network density, average degree, and modularity to determine how well the generated data captured the relationships between microbial communities. These processes were implemented using libraries such as numpy, pandas, torch, scipy, sklearn, and networkx.

### 4.10 Comparison Methods

**FEAST**: FEAST is an expectation‐maximization‐based method that estimates the proportion of the sink community contributed by various source environments [36]. For benchmarking, we employed the R package implementation of FEAST.

**EXPERT**: EXPERT is an ontology‐aware neural network method that leverages transfer learning for microbial community classification [23]. We benchmarked EXPERT using its Python package.

**DeepPhylo**: DeepPhylo is a phylogeny‐informed deep learning model for microbial phenotype and host trait prediction [35]. We used its official GitHub implementation for performance comparison on classification tasks.

**DKI**: DKI is a deep learning‐based approach designed to identify keystone species in microbial communities [43]. We utilized the scripts provided in DKI's GitHub repository to validate the keystone microbes identified by MGM.

**Other Machine Learning Methods**: Additional machine learning models used for benchmarking, including K‐Nearest Neighbor, Logistic Regression, and Random Forest, were implemented using the scikit‐learn library [54].

## Author Contributions

H.Z. and K.N. conceived of and proposed the idea, designed and developed the framework. H.Z., Y.Z., and Z.K. performed the experiments, analyzed the data, and visualized the data. R.Y. provided valuable computing resources. H.Z., Y.Z., Z.K., J.X., R.Y., and K.N. contributed to editing and proof‐reading the manuscript. All authors read and approved the final manuscript. H.Z., Y.Z., and Z.K. contributed equally to this work.

## Conflicts of Interest

The authors declare no conflicts of interest.

## Code Availability

The code for MGM model is available at https://github.com/HUST‐NingKang‐Lab/MGM.

## Supporting information




**Supporting File**: advs74055‐sup‐0001‐SuppMat.docx.

## Data Availability

The Microcorpus‐260K dataset was generated using all samples available from MGnify up to June 2023. It can be downloaded from OneDrive (https://1drv.ms/f/c/18d7815ed643fcef/EhpVq8yLORVImPIqpsFtHa4BrllBymIa‐OWc7jS2vyADzw?e = AzZiLf) following the usage instructions provided at https://github.com/HUST‐NingKang‐Lab/MGM. The cross‐regional cohort was obtained from Clooney et al. (accession number PRJNA414072), and the infant cohort was sourced from Roswall et al. (accession number PRJEB38986). The TCMA dataset is publicly available at https://tcma.pratt.duke.edu. Abundance tables for the GMrepo dataset can be retrieved from the GMrepo database (https://gmrepo.humangut.info/home). The data generated by MGM is available at dx.doi.org /10.6084/m9.figshare.28448000.
