# Supplementary material for: MGM as a Large‐Scale Pretrained Foundation Model for Microbiome Analyses in Diverse Contexts
Source: Adv Sci (Weinh). 2026 Jan 25;13(24):e13333. doi: 10.1002/advs.202513333 (PMC13116254; doi:10.1002/advs.202513333)
Supplement: Supplementary file 1 — Supporting File: advs74055‐sup‐0001‐SuppMat.docx. [file ADVS-13-e13333-s001.docx]

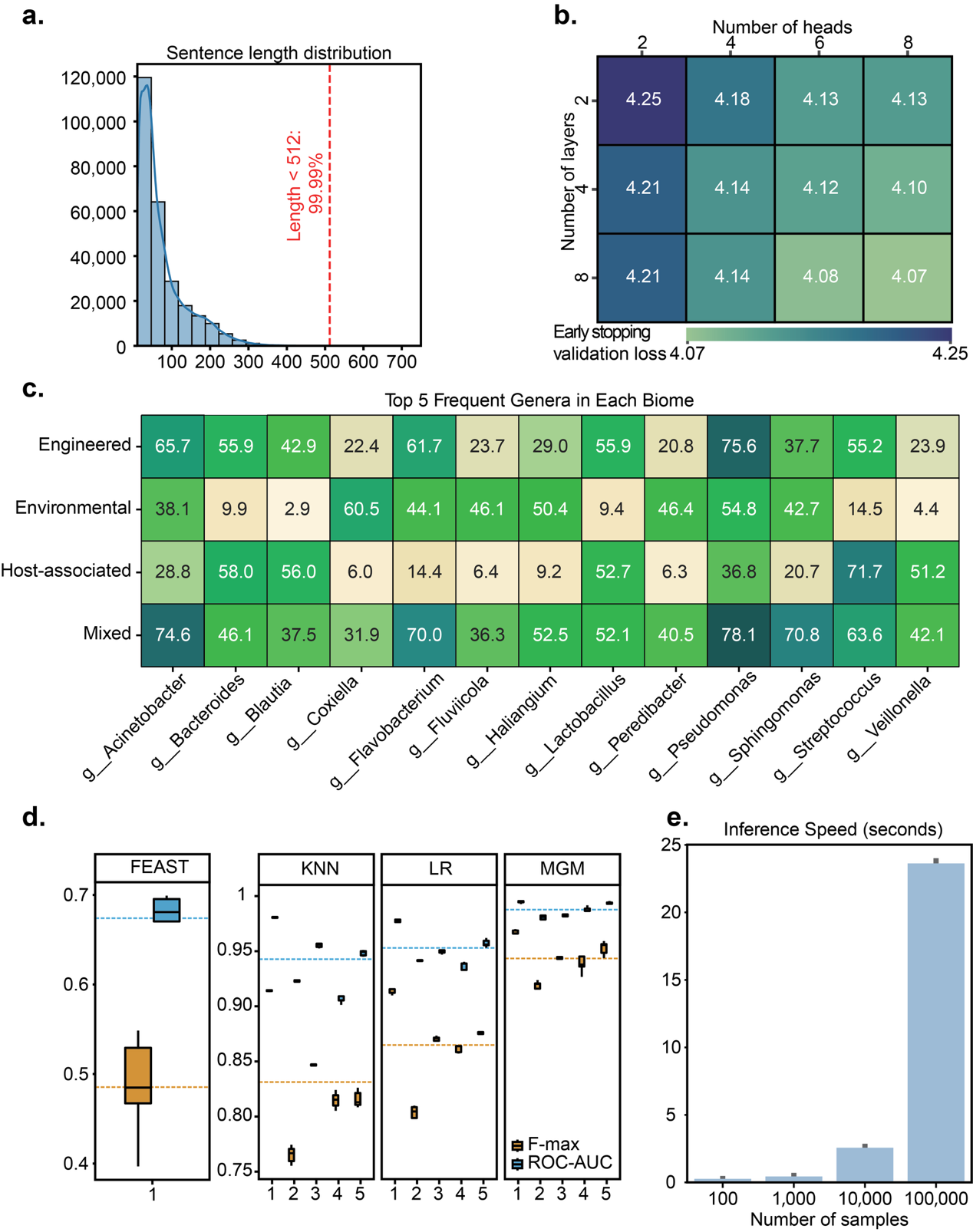


**Supplementary Figure 1. Overview of Dataset Characteristics, Model Optimization, and Evaluation Benchmarks. a.** Distribution of input sequence lengths across samples in MicroCorpus-260K. The red dashed line indicates the fixed input length of 512 tokens. **b.** Early stopping validation loss across different grid search configurations. Rows indicate the number of transformer layers; columns indicate the number of attention heads per layer. During pretraining, a batch size of 64 was used, and validation loss was monitored every 500 steps. Training was stopped if no improvement was observed for five consecutive evaluations. **c.** Heatmap showing the prevalence rate of top 5 most frequent genera across different biomes. **d.** Boxplot comparison of machine learning methods using 5-fold cross-validation across biome lineage levels. Blue bars represent ROC-AUC, orange bars represent F-max, and dashed lines indicate the average performance across all experiments. KNN: K-nearest neighbors; LR: Logistic regression; **e.** Inference speed benchmark showing average runtime on randomly selected subsets of 100, 1,000, 10,000, and 100,000 samples from MicroCorpus-260K. Each setting was repeated five times.


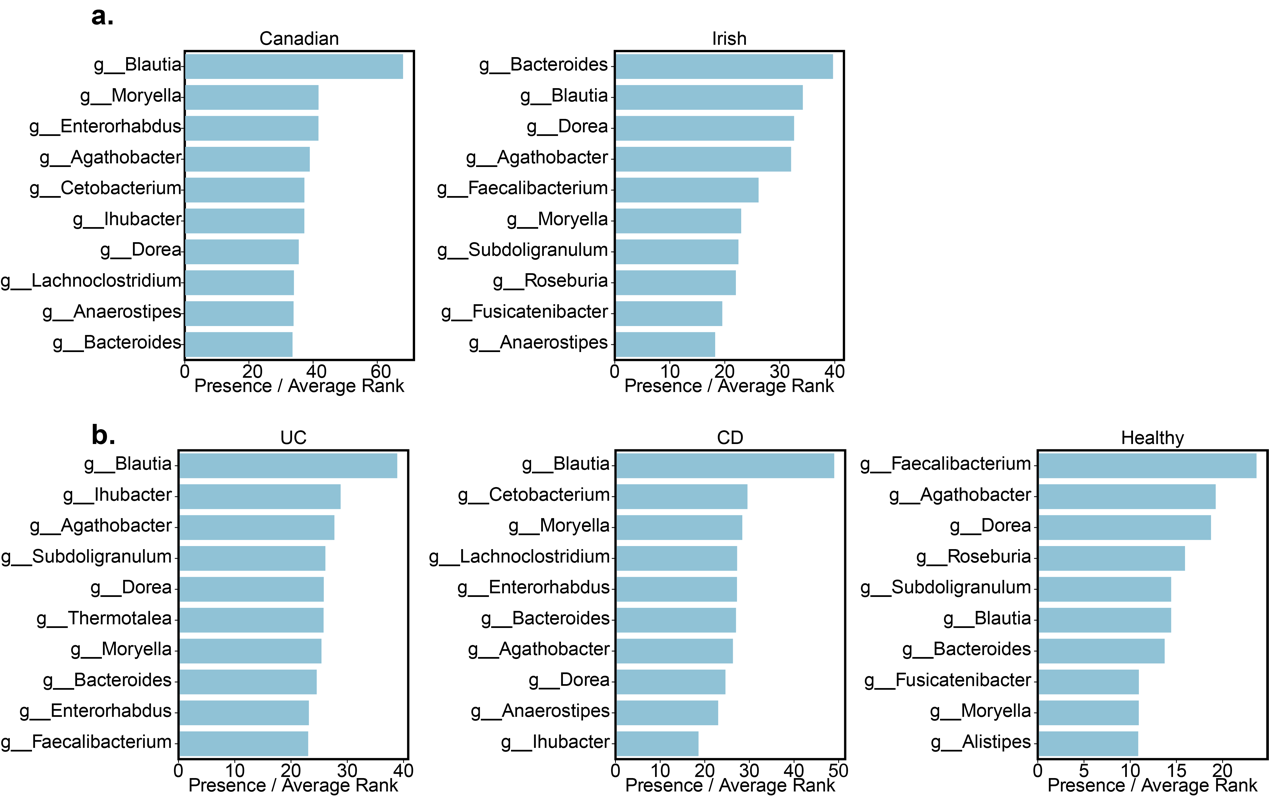


**Supplementary Figure 2. Candidate genus of regions and diseases of IBD cohort. a.** Top 10 Candidate genera of different regions. **b.** Top 10 Candidate genera of different diseases. Candidate genera are measured by their number of occurrences divided by the average rank in each group.

**
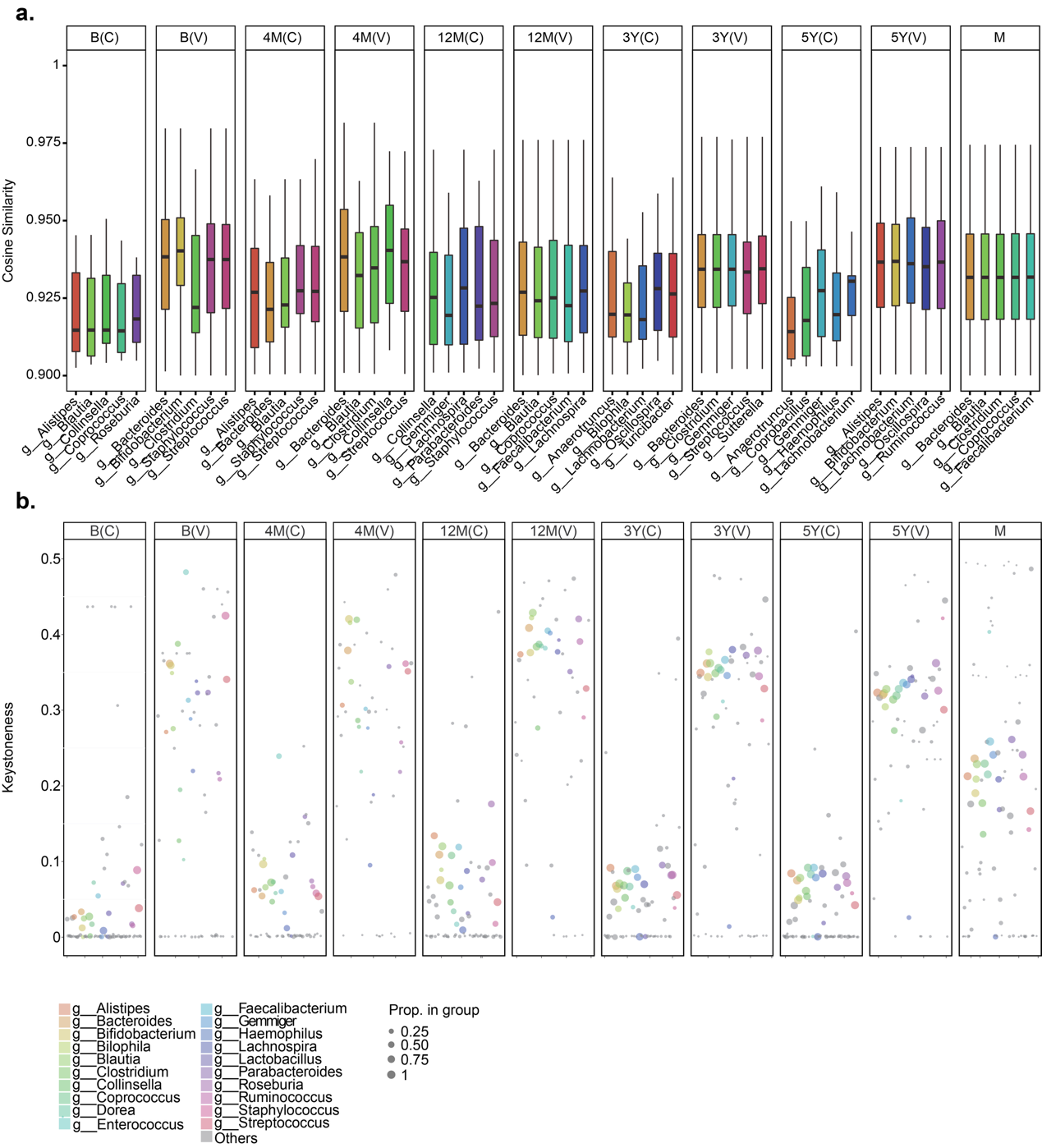
**

**Supplementary Figure 3. Excavation of keystone genera on infant datasets. a.** Top 5 genera with highest deleterious effects in each development stage. **b.** Keystoness of genera with highest attention weights. Top 20 genera with highest attention weights are colored. The size of point represents the proportion that occurs in each development stage.


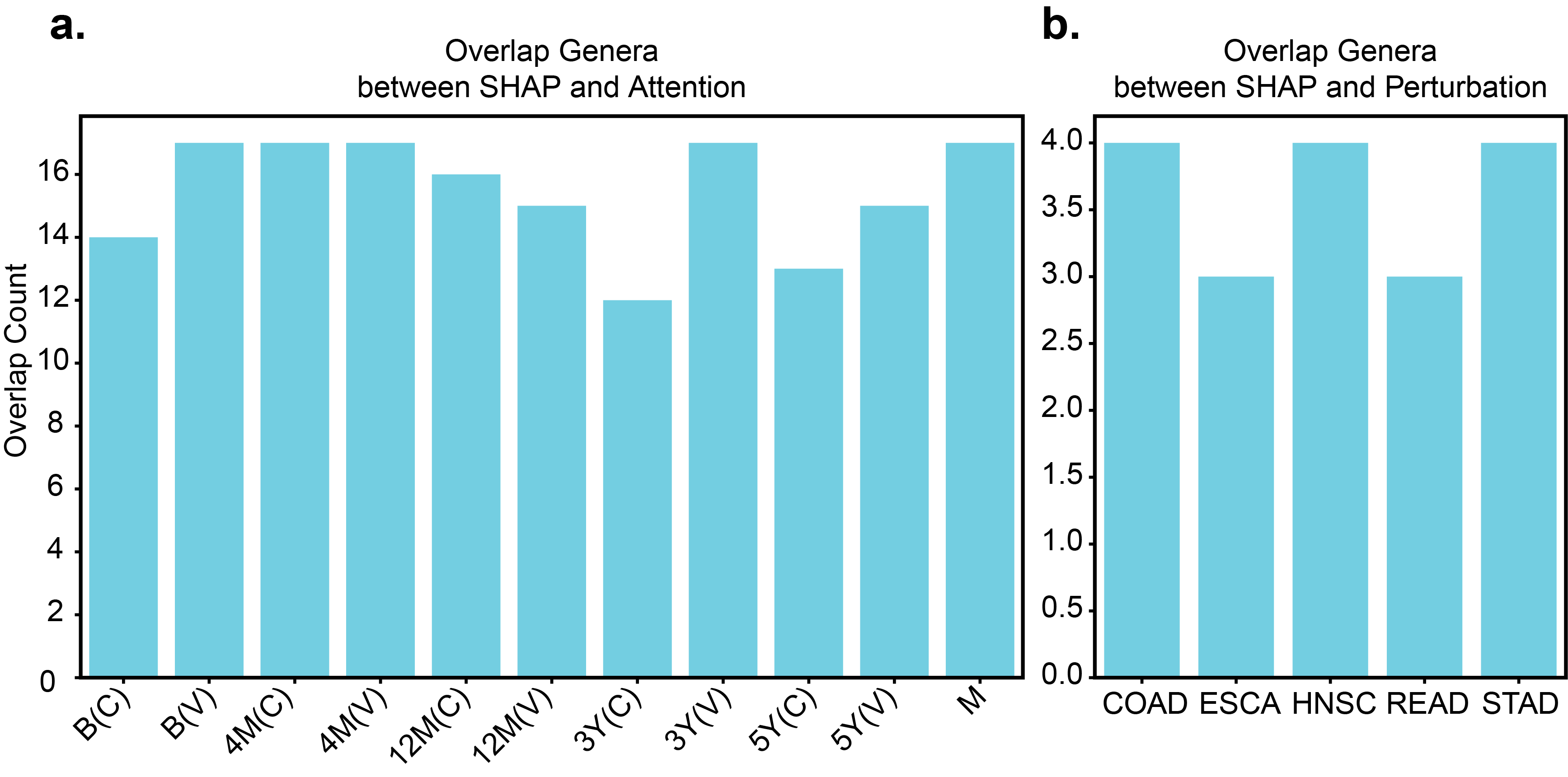


**Supplementary Figure 4. Overlap of important genera identified by MGM and SHAP. a.** Overlap between the top 20 genera ranked by MGM attention scores and SHAP values in the infant cohort. **b.** Overlap between the top 5 genera with the highest deleterious effects (as identified by MGM perturbation analysis) and top SHAP-ranked genera in the TCMA dataset.


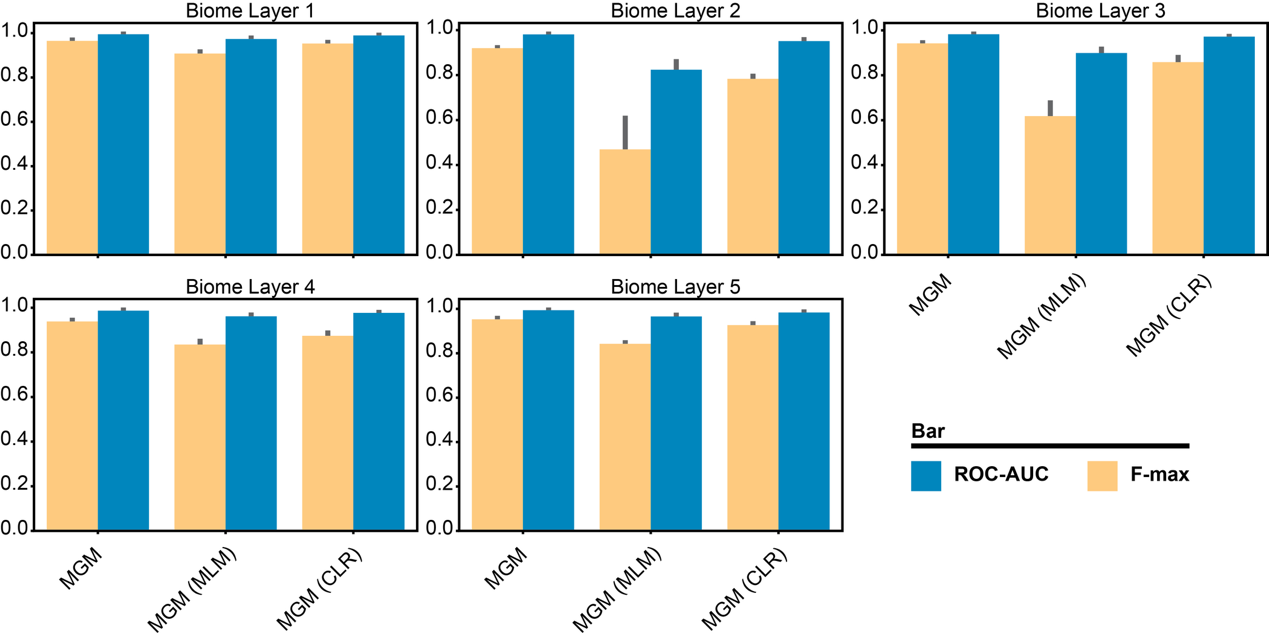


**Supplementary Figure 5.** **Evaluation of encoding strategies and pretraining approaches for microbial community classification.**
